# Navigating Pain Relief: A Comprehensive Review of Transversus Abdominis Plane Block

**DOI:** 10.7759/cureus.51119

**Published:** 2023-12-26

**Authors:** Angan Ghosh, Sanjot Ninave

**Affiliations:** 1 Anaesthesia, Jawaharlal Nehru Medical College, Wardha, IND

**Keywords:** enhanced recovery after surgery (eras), surgical analgesia, pain management, regional anesthesia, transversus abdominis plane block

## Abstract

This comprehensive review explores the multifaceted role of the transversus abdominis plane (TAP) block in contemporary pain management. Beginning with a definition and historical evolution, the article elucidates the mechanism of action, emphasizing local anesthesia, interference with pain signal transmission, and its impact on visceral and somatic pain. The review systematically investigates the diverse indications for TAP block, ranging from its applications in various surgical procedures to postoperative pain management and chronic pain conditions. Noteworthy abdominal wall block variations, including rectus sheath block and quadratus lumborum block, underscore the adaptability of TAP block in diverse clinical scenarios. The implications for clinical practice highlight its pivotal role in enhancing recovery after surgery, reducing opioid reliance, and providing patient-centered care. Furthermore, the article outlines recommendations for further research, addressing ongoing trials, technological innovations, and potential expansions into non-surgical settings. In conclusion, TAP block emerges as a dynamic and indispensable tool in pain management, potentially redefining paradigms and optimizing patient outcomes across a spectrum of medical contexts.

## Introduction and background

The transversus abdominis plane (TAP) block has emerged as a significant intervention in the field of pain management, offering targeted relief for a spectrum of surgical and chronic pain conditions [1]. It is a regional anesthesia technique designed to anesthetize the nerves supplying the anterior abdominal wall by delivering local anesthetic agents into the TAP. This procedure, introduced in the early 2000s, represents a crucial advancement in perioperative and postoperative pain management, particularly for abdominal surgeries. Understanding the intricacies of this technique is pivotal for clinicians seeking to optimize pain relief strategies [2].

A historical perspective is essential to comprehend the current state of the TAP block. The roots of this technique can be traced back to the pursuit of refining abdominal pain management strategies. As we explore the evolution of TAP block, from its conceptualization to its incorporation into contemporary clinical practice, we gain insights into the motivations and innovations that have shaped its trajectory [3].

This review aims to comprehensively analyze the TAP block, encompassing its anatomical underpinnings, mechanisms of action, indications, techniques, clinical efficacy, safety profile, and future directions. By synthesizing existing knowledge and evidence, we aim to offer clinicians, researchers, and healthcare practitioners an up-to-date and consolidated resource for informed decision-making and further exploration in pain management. Through an in-depth exploration of each facet, this review aims to elucidate the various aspects of TAP block, fostering a deeper understanding of its role in contemporary medical practices. As we navigate through the subsequent sections, we will delve into the intricacies of the TAP block, shedding light on its applications, successes, challenges, and potential avenues for future research and innovation.

## Review

Mechanism of action of TAP block

Local Anesthesia and Analgesia

The essence of the TAP block lies in the meticulous and targeted administration of local anesthetic agents into the TAP, a strategically defined anatomical space within the abdominal wall. This sophisticated technique selectively numbs the nerves that transmit pain signals from the abdominal wall. The TAP block is a fundamental and indispensable element in achieving local anesthesia by inducing a reversible sensory blockage. This process effectively alleviates the immediate discomfort associated with surgical incisions and mitigates the trauma experienced during the postoperative period [4].

The localized and targeted effect of the TAP block holds pivotal significance in enhancing patient comfort, providing a more tolerable and less painful recovery experience. By precisely numbing specific nerve pathways, the technique contributes to a pronounced reduction in immediate postoperative pain, fostering an environment conducive to smoother recuperation. Furthermore, this enhanced comfort empowers patients to engage in early mobilization, a key aspect of recovery [4].

The active facilitation of early mobilization with the TAP block is particularly noteworthy, aligning seamlessly with contemporary healthcare goals and prioritizing optimizing postoperative outcomes and overall patient well-being. The combination of targeted pain relief and encouragement of early movement positions the TAP block as a progressive and patient-centric intervention within perioperative care [4].

Interference with Pain Signal Transmission

The significance of the TAP block extends beyond conventional local anesthesia, focusing on a sophisticated mechanism that intricately disrupts the transmission of pain signals within the TAP. Rather than operating on the neuroaxis, the TAP block strategically modulates pain perception by actively intervening in the propagation of nociceptive signals originating from the surgical site to the peripheral nervous system [5]. This intricate interference occurs at the peripheral nerve level, constituting a pivotal component in the comprehensive strategy of perioperative pain management. By selectively impeding the transmission of pain signals, the TAP block emerges as a critical and strategic player in the immediate postoperative period, fundamentally contributing to the minimization of both the intensity and duration of pain experienced by patients during this crucial phase of recovery [5].

Particularly in the initial postoperative period, when patients are most susceptible to acute pain, the TAP block's ability to actively modulate pain perception contributes significantly to the overall enhancement of the patient's comfort and recovery experience. The selective interruption of nociceptive signals represents a targeted approach that aligns with contemporary goals of optimizing postoperative outcomes and prioritizing patient well-being [6]. In essence, the TAP block serves as a sophisticated intervention beyond mere pain relief; it actively shapes the patient's postoperative journey by intricately disrupting pain signalling mechanisms, thereby contributing to a more comfortable and expedited recovery [5].

Impact on Visceral and Somatic Pain

The TAP block stands out with a distinguishing characteristic: its remarkable capacity to effectively manage both visceral and somatic pain. In the intricate landscape of abdominal surgeries, where visceral pain stems from internal organs and somatic pain originates from musculoskeletal structures, these two types of pain often coexist. The TAP block's multifaceted impact seamlessly extends to both domains, engendering a comprehensive and synergistic analgesic effect [5]. This dual benefit becomes particularly invaluable in the context of surgical procedures featuring midline abdominal incisions, a setting where the convergence of visceral and somatic pain is notably prevalent. The TAP block is pivotal in this scenario by simultaneously addressing these distinct pain modalities. By effectively managing both visceral and somatic pain, the TAP block emerges as a versatile and integral component in perioperative pain management strategies [6]. The TAP block's contribution goes beyond traditional pain relief; it becomes a linchpin in promoting the holistic comfort and well-being of the patient throughout the recovery period. The ability to comprehensively address diverse pain experiences enhances the patient's postoperative journey and aligns with contemporary principles of patient-centered care. The TAP block's dual impact on visceral and somatic pain positions it as an adaptable and indispensable tool, embodying the commitment to optimizing the overall patient experience during recovery [6].

Indications for TAP block

Surgical Applications

Abdominal surgeries: A primary and well-established domain showcasing the efficacy of the TAP block within the realm of abdominal surgeries is essential for comprehensive understanding and implementation. Precision is a hallmark of this technique as it targets the nerves innervating the anterior abdominal wall, minimizing perioperative pain. Its versatile application spans a spectrum of abdominal procedures, including but not limited to appendectomies, hernia repairs, and colorectal surgeries. The TAP block's localized and targeted approach aligns seamlessly with the imperative need for focused analgesia in these surgical contexts, contributing significantly to heightened patient comfort and facilitating an expedited postoperative recovery. The versatility exhibited by the TAP block positions it as a valuable asset in the arsenal of techniques dedicated to optimizing outcomes in diverse abdominal surgeries [4].

Obstetric and gynecological surgeries: Beyond its role in general abdominal surgeries, the utility of the TAP block extends meaningfully into obstetric and gynecological procedures, notably exemplified in scenarios such as cesarean sections and hysterectomies. The TAP block's unique capacity to deliver effective pain relief in these specific settings enhances maternal satisfaction and promotes early ambulation, contributing to an overall positive birthing experience. The adaptability of the TAP block extends its impact to diverse surgical contexts within obstetric and gynecological care, emphasizing its role as a versatile and patient-focused technique. This underscores the broad applicability of the TAP block in enhancing postoperative recovery experiences across a spectrum of surgical interventions within the abdominal and pelvic regions [7].

Postoperative Pain Management

In postoperative pain management, the TAP block is pivotal and strategic in implementing Enhanced Recovery After Surgery (ERAS) protocols. Its primary objective of mitigating postoperative pain seamlessly aligns with the fundamental tenets of ERAS, embodying a multifaceted approach to perioperative care. The TAP block actively supports critical components integral to ERAS protocols, including but not limited to early mobilization, reduction in opioid consumption, and expeditious recovery [7]. Functioning as a cornerstone in multimodal analgesia strategies, the TAP block is integral in optimizing postoperative outcomes across diverse surgical specialties. Its inclusion in ERAS protocols signifies a commitment to patient-centered care, underscoring a comprehensive and proactive approach to pain management that transcends traditional paradigms. The TAP block is instrumental in fostering a more patient-friendly and efficient recovery process by actively contributing to reducing postoperative pain and its associated challenges. Incorporating TAP block within ERAS protocols reflects a contemporary and patient-centric paradigm, emphasizing enhancing the overall postoperative experience and outcomes [8].

Chronic Pain Conditions

In chronic pain management, the TAP block emerges as a promising and effective intervention for conditions characterized by abdominal wall pain. Chronic pain syndromes, notably anterior cutaneous nerve entrapment syndrome (ACNES) and postsurgical neuropathic pain, frequently manifest within the abdominal region. The TAP block, distinguished by its precise and targeted approach, is valuable in addressing and alleviating the persistent discomfort associated with these chronic pain conditions [8]. The TAP block's capacity to provide targeted relief in the abdominal wall region is a noteworthy addition to the broader landscape of pain management for chronic conditions. By specifically targeting the nerves implicated in abdominal wall pain syndromes, the TAP block contributes to the comprehensive arsenal of interventions aimed at improving the quality of life for individuals grappling with chronic pain. This underscores the adaptability of the TAP block beyond its traditional role in perioperative care, positioning it as a versatile and valuable tool in the multifaceted and evolving field of chronic pain management [9].

Techniques of performing TAP block

Ultrasound-Guided TAP Block

Ultrasound-guided TAP block represents a sophisticated approach to regional anesthesia, leveraging real-time visualization through ultrasound imaging. The principle of this technique involves the dynamic visualization of the layers of the abdominal wall, particularly the TAP, ensuring precision in the deposition of the anesthetic agent. The key advantages of employing ultrasound guidance in TAP block are twofold. Firstly, it significantly enhances the safety of the procedure by allowing for the clear identification of crucial anatomical structures such as blood vessels, minimizing the risk of inadvertent vascular puncture. Secondly, ultrasound guidance tailors the block to the individual patient's anatomy, ensuring a more personalized and practical approach to pain management [10].

The ultrasound-guided TAP block entails a methodical procedure designed to enhance precision and effectiveness. Beginning with patient positioning in a supine or slightly lateral orientation, this setup ensures optimal accessibility to the abdominal wall for the ensuing steps. The ultrasound probe placement is a critical phase, strategically positioned to capture a clear and detailed image of the abdominal wall layers, focusing on identifying the TAP. This real-time imaging serves as a cornerstone for procedural precision. Subsequently, needle insertion becomes a carefully executed step, guided continuously by ultrasound, ensuring accurate advancement into the TAP. This crucial phase aims to deliver the local anesthetic agent precisely, avoiding unintended structures and optimizing the desired analgesic effect. The confirmation step is integral, involving the observation of the real-time spread of the local anesthetic within the TAP on the ultrasound screen. This immediate feedback mechanism verifies the proper needle placement and allows for real-time adjustments, exemplifying the commitment to procedural accuracy and patient safety [10].

Landmark-Based TAP Block

The landmark-based TAP block operates on the principles of needle placement guided by anatomical landmarks, eliminating the need for real-time imaging guidance. While this approach may be more straightforward, its accurate execution demands a comprehensive understanding of abdominal wall anatomy. This technique is precious in situations where ultrasound equipment is unavailable or when the operator possesses a high level of proficiency in identifying anatomical landmarks. The reliance on palpable structures necessitates a meticulous understanding of the patient's anatomy for successful implementation [11].

The procedural steps of the landmark-based TAP block follow a structured sequence guided by anatomical references. Initiated by identifying critical anatomical landmarks, such as the iliac crest, costal margin, and anterior superior iliac spine, these palpable structures serve as crucial reference points for precise needle placement, facilitating the localization of the TAP. Subsequently, the needle is carefully directed through the skin and underlying tissues, guided by the identified anatomical landmarks, to reach the target plane. The importance of precision in needle placement cannot be overstated, as it is pivotal for ensuring optimal efficacy and safety. Following needle insertion, the procedural course includes the performance of aspiration to confirm the avoidance of blood vessels and verify accurate needle placement. Once this confirmation is secured, the anesthetic agent is injected into the TAP, instigating the desired analgesic effect. This structured approach underscores the reliance on anatomical landmarks without real-time imaging, showcasing the adaptability of the landmark-based TAP block procedure in various clinical contexts [12].

Choice of Injectate and Dosage

Selection of local anesthetic: In the TAP block, the choice of local anesthetic emerges as a pivotal consideration with far-reaching implications. Among the commonly employed agents are bupivacaine, ropivacaine, and lidocaine, each with its unique pharmacological profile. The decision on which local anesthetic to administer hinges on several factors, prominently the anticipated duration of analgesia and the patient's medical history. Bupivacaine, known for its prolonged action, might be favored in scenarios requiring extended postoperative pain control. At the same time, lidocaine, with its rapid onset, could be selected for procedures demanding a shorter duration of anesthesia. The selection process demands a nuanced understanding of the patient's health, the surgical context, and the desired balance between duration and onset of analgesia [12].

Dosage considerations: Tailoring the dosage of the chosen local anesthetic is a meticulous process intricately woven into the fabric of the specific clinical scenario and the nuanced characteristics of the individual patient. Several factors come to the forefront, influencing the dosage decision-making process. The type of surgery performed plays a pivotal role, with more extensive procedures often necessitating a higher dosage for effective pain relief. Patient age and comorbidities contribute additional layers of complexity, as the physiological response to the local anesthetic may vary. Moreover, the desired duration of pain relief becomes a crucial determinant, guiding the dosage to align with the anticipated duration of the postoperative or post-procedural pain. A delicate balance is sought, where careful attention to dosage optimizes analgesic efficacy and minimizes systemic toxicity risk. This nuanced approach to dosage consideration underscores the commitment to personalized and patient-centered pain management in TAP block, acknowledging the diversity of clinical scenarios and patient profiles [13].

Clinical efficacy and outcomes

Evidence from Clinical Trials

Numerous studies have rigorously investigated the clinical efficacy of the TAP block in the context of abdominal surgery, offering valuable insights into its impact on postoperative outcomes. A comprehensive systematic review and meta-analysis of randomized controlled trials concluded that the TAP block not only exhibited a high level of safety but also significantly reduced postoperative morphine requirements and mitigated occurrences of nausea and vomiting [14]. Building upon these findings, an additional study observed a noteworthy reduction in morphine consumption at the 24-hour postoperative mark among patients who received the TAP block [15]. Further exploration into specific surgical scenarios revealed promising results. A systematic review identified enhanced analgesia in patients undergoing various abdominal procedures such as laparotomy for colorectal surgery, laparoscopic cholecystectomy, and both open and laparoscopic appendectomy [16]. Notably, the review indicated a trend towards superior analgesic outcomes when utilizing 15 mL or more of local anesthetic per side compared to lesser volumes.

However, amidst these positive findings, the review highlighted a certain degree of inconsistency in results from comparative studies on TAP block. This discrepancy suggests lingering questions regarding the nuanced effects of factors such as the type of surgical procedure, block dosage, block technique, and block timing on TAP block analgesia [16]. Although most reviewed trials lean towards superior early pain control, the precise identification of optimal surgical procedures, dosages, techniques, and timing that provide the most effective analgesia remains elusive [16]. In essence, while the TAP block shows great promise in reducing postoperative pain and minimizing opioid requirements, the complexity of these variables underscores the need for further dedicated research. There is a compelling call for more comprehensive investigations to delineate the precise clinical efficacy and the optimal application of TAP block in diverse surgical contexts. As the field advances, ongoing research efforts are crucial for refining our understanding and harnessing the full potential of TAP block in enhancing perioperative care [16].

Comparison with Other Pain Management Techniques

Numerous studies have compared the analgesic efficacy of the TAP block with other pain management techniques, shedding light on its potential advantages in specific contexts. A systematic review and meta-analysis compared continuous TAP block with epidural analgesia (EA) in adults post-abdominal surgery. The findings indicated that TAP block was associated with lower pain scores and reduced opioid consumption, positioning it as a favorable alternative to EA [17]. Similarly, another study comparing TAP block with EA found that TAP block demonstrated non-inferiority in terms of pain control and opioid consumption, further supporting its viability as an effective analgesic intervention [18]. A specific investigation focused on patients undergoing open appendicectomy reaffirmed the efficacy of TAP block, showcasing significantly reduced morphine consumption 24 hours post surgery [15].

Expanding the comparative scope, a systematic review compared TAP block with wound infiltration (WI) for postoperative pain management in abdominal surgery. The review concluded that TAP block was associated with superior analgesia and decreased opioid consumption compared to WI [19]. However, determining the optimal pain management technique is nuanced and may rely on factors such as the specific surgical procedure and patient characteristics. Hence, there remains a need for further research to delineate the most effective approach for different contexts, acknowledging the diversity inherent in surgical scenarios and patient profiles [19]. These comparative studies contribute valuable insights to the evolving landscape of perioperative pain management, guiding clinicians in making informed decisions tailored to individual patient needs and procedural requirements.

Patient-Reported Outcomes and Satisfaction

Several studies have delved into patient-reported outcomes and satisfaction in the context of the TAP block, shedding light on its impact on the overall postoperative experience. While reporting mixed findings, a systematic review and meta-analysis highlighted that the TAP block improved patient satisfaction in two trials, albeit with a reduction in satisfaction observed in one trial [7]. This nuanced perspective suggests that the impact of TAP block on patient satisfaction may be influenced by various factors, warranting a closer examination. In a comparative study, TAP block was pitted against WI in adult patients undergoing surgery, with the results indicating higher patient satisfaction with TAP block [5]. Furthermore, a specific investigation comparing preemptive and postoperative ultrasound-guided bilateral TAP block after laparoscopic cholecystectomy demonstrated increased patient satisfaction associated with TAP block usage [20]. These findings suggest a positive trend wherein TAP block enhances patient satisfaction in postoperative pain management. However, it is crucial to acknowledge the complexity of patient-reported outcomes and satisfaction, which individual variations and diverse surgical contexts can influence. While existing studies provide valuable insights, there is a recognized need for further research to comprehensively assess patient satisfaction across various surgical scenarios and patient populations. This ongoing exploration is essential for refining our understanding of the nuanced interplay between TAP block and patient-reported outcomes, ultimately informing clinical practices and optimizing the postoperative experience for diverse patients [7,20].

Safety and complications

Common Side Effects

Localized pain and bruising: Following a TAP block, patients may experience transient discomfort at the injection site, a common and typically mild side effect. This localized pain is generally self-limiting, gradually alleviating over time. Additionally, bruising at the needle insertion point is a frequent occurrence, resulting from inadvertent trauma to blood vessels during the procedure. While localized pain and bruising are generally benign and temporary, they emphasize the importance of effective patient communication and expectation management during the post-TAP block period. Providing patients with clear information about these anticipated side effects aids in their understanding and contributes to a more informed and reassured recovery [21].

Temporary motor block: A notable side effect of TAP block is the transient motor block experienced in the abdominal muscles. This temporary weakness or motor block arises from the diffusion of the local anesthetic to motor nerves in the vicinity. While this effect is expected to be short-lived, resolving as the anesthetic gradually wears off, healthcare providers must communicate this aspect to patients. Informing patients about the anticipated post-procedural sensations facilitates a smoother recovery experience and helps manage expectations regarding temporary changes in abdominal muscle function [22].

Nausea and vomiting: Post TAP block, especially following abdominal surgeries, patients may contend with symptoms of nausea and vomiting. While the etiology of these symptoms is often multifactorial, TAP block itself may contribute to their occurrence. Addressing and managing nausea and vomiting commonly involves the implementation of antiemetic measures, contributing to the overall comfort and well-being of patients undergoing TAP block. Proactive management of these symptoms enhances the postoperative experience, aligning with patient-centered care and promoting a more favorable recovery period [23].

Rare Complications

Systemic toxicity: While exceedingly rare, systemic toxicity poses a potential but severe risk associated with the TAP block, mainly if there is inadvertent intravascular injection of the local anesthetic. This rare occurrence can result in central nervous system and cardiovascular manifestations, such as seizures and cardiac arrhythmias. However, stringent adherence to proper injection techniques, including aspiration before injection, and careful dosage considerations significantly mitigate the risk of systemic toxicity. Clinicians, therefore, play a pivotal role in ensuring patient safety by maintaining a meticulous approach to TAP block administration, reducing the likelihood of such rare but severe complications [24].

Infection: Although infrequent, infection at the injection site remains a potential complication of TAP block. Rigorous adherence to aseptic techniques during the procedure minimizes the risk of introducing pathogens. By maintaining a sterile environment and employing comprehensive infection prevention measures, healthcare providers contribute to preventing infection-related complications, emphasizing the importance of procedural hygiene in the overall safety of TAP block [25].

Vascular injury: Vascular injury, including hematoma formation, represents a rare but conceivable complication that may arise during needle insertion in TAP block. Heightened awareness of vascular anatomy and meticulous needle placement are critical in reducing the likelihood of vascular complications. Clinicians must thoroughly understand the abdominal vascular structures to navigate potential risks effectively. By prioritizing precision and attentiveness during needle insertion, healthcare providers can minimize vascular injuries, highlighting the importance of anatomical knowledge and procedural skills in safely executing TAP block [26].

Patient Selection and Risk Assessment

Pre-procedural assessment: A thorough pre-procedural assessment is a foundational pillar before undertaking a TAP block, emphasizing the significance of understanding the patient's medical history and current health status. Essential factors, including pre-existing medical conditions, allergies, and the history of prior surgeries, necessitate careful consideration. A focused abdominal examination is an integral component of this assessment, aiding in identifying contraindications or anatomical variations that might impact the procedural approach. This meticulous evaluation sets the stage for a tailored and patient-centered TAP block procedure [27-28].

Risk-benefit analysis: Conducting an individualized risk and benefit assessment is an imperative step in patient selection for the TAP block. The potential benefits of TAP block in delivering effective pain relief must be carefully weighed against the patient's overall health status and the potential for procedural complications. This nuanced analysis guides clinicians in making decisions tailored to each patient's circumstances. By considering the anticipated advantages and potential risks, healthcare providers ensure that the decision to proceed with the TAP block aligns with patient-centered care and safety principles. This risk and benefit assessment becomes a pivotal component of the shared decision-making process between healthcare providers and patients [29].

Variations and modifications of TAP block

Rectus Sheath Block

Anatomy and rationale: A noteworthy modification to the traditional TAP block involves the integration of a rectus sheath block. This adaptation introduces a local anesthetic between the rectus abdominis muscle and its sheath. This modification specifically targets the anterior cutaneous branches of the intercostal nerves, aiming to provide additional analgesic coverage to the midline abdominal wall. The rationale behind combining a TAP block with a rectus sheath block is rooted in the aspiration for more comprehensive analgesia, particularly in surgical scenarios involving midline incisions such as laparotomies. This strategic modification seeks to address pain emanating from the midline structures, offering an enhanced and targeted approach to pain relief [4].

Clinical applications: The clinical applications of augmenting TAP block with a rectus sheath block are particularly pronounced in procedures like midline laparotomies, where visceral and somatic pain pathways converge. By extending the reach of analgesic coverage to the midline structures, this modification contributes to a more holistic pain management strategy. The potential benefits extend beyond immediate postoperative pain relief, impacting opioid consumption and potentially fostering an expedited recovery for the patient. This combined approach reflects a nuanced understanding of the intricacies of pain pathways, showcasing the adaptability of TAP block to different surgical contexts and the commitment to refining analgesic strategies for optimal patient outcomes [30].

Quadratus Lumborum Block

Anatomy and mechanism: Integrating the quadratus lumborum block as an adjunct to the TAP block introduces a distinctive modification in enhancing pain management. The quadratus lumborum muscle, situated posterolateral to the transversus abdominis muscle, becomes the focal point of this modification. By targeting the quadratus lumborum, the sensory blockade achieved extends beyond the confines of the anterior abdominal wall. This strategic modification addresses the challenge of posterior abdominal pain, aiming to provide a more thorough analgesic effect. The anatomical precision in targeting the quadratus lumborum reflects a nuanced understanding of the sensory innervation in the abdominal region. It underscores the adaptability of the TAP block to diverse clinical scenarios [31].

Applications and benefits: Integrating the quadratus lumborum block as an adjunct to the TAP block has manifested notable efficacy, particularly in surgical procedures involving the posterior abdominal wall. Renal surgeries and spine procedures, where manipulation and intervention in the posterior abdominal structures are commonplace, are prime examples. This modification significantly extends the reach of the TAP block, offering a more encompassing coverage of sensory innervation in the abdominal region. The tangible benefits of combining quadratus lumborum block with TAP block contribute to a multimodal approach to pain management. By addressing the anterior and posterior components of the abdominal wall, this combined technique aligns with the contemporary emphasis on personalized and comprehensive pain relief strategies. Integrating the quadratus lumborum block into the TAP block exemplifies a tailored approach to pain management, acknowledging the diverse anatomical challenges posed by different surgical contexts and enhancing the overall quality of perioperative care [32].

Combining TAP Block with Other Techniques

Synergy and comprehensive analgesia: Recognizing the synergistic potential inherent in regional anesthesia techniques, clinicians have strategically explored combining the TAP block with other nerve blocks to achieve a more extensive and prolonged analgesic effect. Notably, the amalgamation of TAP block with EA or thoracic paravertebral blocks has been scrutinized in specific surgical scenarios. This combination approach strategically leverages the distinctive strengths of each technique, forming a symbiotic relationship that culminates in a balanced and nuanced approach to pain management. By synergistically capitalizing on the targeted benefits of different nerve blocks, clinicians endeavor to enhance the quality and duration of analgesia while minimizing the reliance on systemic opioids, thus contributing to an optimized postoperative recovery experience [33].

Tailoring to patient needs: The decision to integrate TAP block with other regional anesthesia techniques is intricately tailored to the unique characteristics of each patient, encompassing their surgical profile, pain requirements, and the inherent nature of the procedure at hand. This deliberate modification reflects regional anesthesia's dynamic and evolving landscape, where a personalized approach to interventions based on individual patient needs is increasingly prioritized. The customization of the pain management strategy acknowledges the diversity among patients, ensuring that interventions are effective and aligned with each individual's specific requirements and preferences. In this way, integrating TAP block with other techniques exemplifies a patient-centric paradigm in regional anesthesia, embodying the contemporary trend towards precision and customization in perioperative care [34].

## Conclusions

Exploring the TAP block unveils a versatile and targeted intervention in pain management. Key findings highlight its efficacy in providing localized pain relief across various surgical scenarios, from abdominal surgeries to obstetric procedures, postoperative care, and even chronic pain conditions. The nuanced mechanisms of action, incorporating local anesthesia, interference with pain signal transmission, and addressing both visceral and somatic pain, underscore the comprehensive approach of TAP block. Variations and modifications, such as rectus sheath and quadratus lumborum blocks, showcase its adaptability to diverse clinical contexts. At the same time, the combination with other techniques signifies a paradigm shift in regional anesthesia strategies. Implications for clinical practice are profound, positioning TAP block as an indispensable tool in enhancing recovery after surgery, reducing opioid use, and promoting patient-centered care. Recommendations for further research underscore the ongoing quest to refine dosages, patient selection criteria, and long-term outcomes. At the same time, technological advancements and the exploration of novel applications in non-surgical settings and chronic pain management continue to shape the dynamic landscape of TAP block. As clinicians and researchers unravel its intricacies, TAP block promises to redefine pain management paradigms and optimize patient outcomes in the evolving landscape of contemporary medicine.
